# Supplementary material for: COVID-19: Tail risk and predictive regressions
Source: PLoS One. 2022 Dec 1;17(12):e0275516. doi: 10.1371/journal.pone.0275516 (PMC9714707; doi:10.1371/journal.pone.0275516)
Supplement: S1 Table — (PDF) [file pone.0275516.s001.pdf]

**Table S1.** Wild bootstrap quasi-differenced unit root tests for Infections based on Rademacher distribution with sieve based recolouring ($p-$values in brackets)

| | $\Delta Infections$ | | | | | | $\Delta^2 Infections$ | | | | | |
|---|---|---|---|---|---|---|---|---|---|---|---|---|
| | $LR$ | $MZ_\alpha$ | $MSB$ | $MZ_t$ | $MP_t$ | $ADF$ | $LR$ | $MZ_\alpha$ | $MSB$ | $MZ_t$ | $MP_t$ | $ADF$ |
| UK | 0.44 | -2.04 | 0.49 | -1.00 | 11.96 | -0.99 | 120.41 | -202.64 | 0.05 | -10.07 | 0.12 | -23.98 |
| | (0.42) | (0.48) | (0.67) | (0.42) | (0.52) | (0.43) | (0.00) | (0.00) | (0.00) | (0.00) | (0.00) | (0.00) |
| Germany | 0.59 | -2.98 | 0.39 | -1.17 | 8.11 | -1.13 | 136.23 | -190.84 | 0.05 | -9.75 | 0.16 | -27.93 |
| | (0.44) | (0.44) | (0.54) | (0.39) | (0.45) | (0.44) | (0.00) | (0.00) | (0.00) | (0.00) | (0.00) | (0.00) |
| France | 0.34 | 7.63 | 0.34 | 2.62 | 25.40 | -0.95 | 147.60 | -171.55 | 0.05 | -8.77 | 0.85 | -31.02 |
| | (0.41) | (1) | (0.23) | (1) | (0.73) | (0.38) | (0.00) | (0.00) | (0.00) | (0.00) | (0.00) | (0.00) |
| Italy | 0.00 | -0.26 | 0.78 | -0.20 | 34.90 | 0.30 | 89.82 | -204.64 | 0.05 | -10.01 | 0.26 | -17.77 |
| | (0.64) | (0.6) | (0.91) | (0.59) | (0.82) | (0.73) | (0.00) | (0.00) | (0.00) | (0.00) | (0.00) | (0.00) |
| Spain | 6.41 | -12.76 | 0.19 | -2.48 | 2.12 | -3.12 | | | | | | |
| | (0.01) | (0.05) | (0.05) | (0.06) | (0.05) | (0.02) | | | | | | |
| Russia | 0.01 | -0.22 | 1.09 | -0.24 | 61.72 | -0.24 | 140.35 | -182.19 | 0.05 | -9.54 | 0.13 | -29.47 |
| | (0.6) | (0.65) | (0.93) | (0.6) | (0.88) | (0.6) | (0.00) | (0.00) | (0.00) | (0.00) | (0.00) | (0.00) |
| Netherland | 0.00 | -0.07 | 0.70 | -0.05 | 30.29 | 0.03 | 94.31 | -194.36 | 0.05 | -9.83 | 0.16 | -19.16 |
| | (0.7) | (0.71) | (0.88) | (0.71) | (0.83) | (0.73) | (0.00) | (0.00) | (0.00) | (0.00) | (0.00) | (0.00) |
| Sweden | 12.97 | -22.88 | 0.15 | -3.38 | 1.07 | -4.37 | | | | | | |
| | (0) | (0.03) | (0.04) | (0.03) | (0.02) | (0.01) | | | | | | |
| India | 0.00 | -0.04 | 0.95 | -0.04 | 50.30 | -0.01 | 132.27 | -193.08 | 0.05 | -9.81 | 0.15 | -26.94 |
| | (0.64) | (0.67) | (0.93) | (0.66) | (0.88) | (0.67) | (0.00) | (0) | (0.10) | (0) | (0) | (0.00) |
| Austria | 0.01 | -0.71 | 0.62 | -0.44 | 22.17 | -0.25 | 90.76 | -194.57 | 0.05 | -9.86 | 0.13 | -18.44 |
| | (0.63) | (0.59) | (0.75) | (0.56) | (0.68) | (0.63) | (0.00) | (0.00) | (0.00) | (0.00) | (0.00) | (0.00) |
| Finland | 0.00 | -0.24 | 0.56 | -0.13 | 21.32 | 0.89 | 137.87 | -187.21 | 0.05 | -9.66 | 0.15 | -28.53 |
| | (0.72) | (0.71) | (0.8) | (0.7) | (0.75) | (0.88) | (0.00) | (0.00) | (0.00) | (0.00) | (0.00) | (0.00) |
| Ireland | 4.71 | -25.97 | 0.14 | -3.60 | 0.96 | -3.36 | | | | | | |
| | (0.02) | (0.03) | (0.03) | (0.03) | (0.03) | (0.03) | | | | | | |
| US | 0.14 | -0.80 | 0.74 | -0.59 | 27.91 | -0.59 | 146.30 | -181.65 | 0.05 | -9.52 | 0.15 | -30.76 |
| | (0.55) | (0.63) | (0.92) | (0.55) | (0.81) | (0.55) | (0.00) | (0.00) | (0.00) | (0.00) | (0.00) | (0.00) |
| Lithuania | 0.30 | -1.42 | 0.59 | -0.83 | 17.01 | -0.82 | 112.89 | -187.92 | 0.05 | -9.69 | 0.13 | -23.35 |
| | (0.49) | (0.54) | (0.77) | (0.47) | (0.65) | (0.49) | (0.00) | (0.00) | (0.00) | (0.00) | (0.00) | (0.00) |
| Canada | 0.30 | -1.42 | 0.59 | -0.83 | 17.01 | -0.82 | 135.25 | -192.72 | 0.05 | -9.79 | 0.17 | -27.59 |
| | (0.49) | (0.54) | (0.77) | (0.47) | (0.65) | (0.49) | (0.00) | (0.00) | (0.00) | (0.00) | (0.00) | (0.00) |
| Brazil | 0.04 | -0.18 | 0.62 | -0.11 | 25.12 | -0.37 | 128.47 | -174.91 | 0.05 | -9.35 | 0.14 | -27.54 |
| | (0.6) | (0.66) | (0.84) | (0.65) | (0.76) | (0.6) | (0.00) | (0.00) | (0.00) | (0.00) | (0.00) | (0.00) |
| Mexico | 0.00 | -1.20 | 0.49 | -0.59 | 14.86 | -0.13 | 128.34 | -173.32 | 0.05 | -9.31 | 0.14 | -27.64 |
| | (0.68) | (0.5) | (0.6) | (0.5) | (0.53) | (0.68) | (0.00) | (0.00) | (0.00) | (0.00) | (0.00) | (0.00) |
| Argentina | 0.62 | -2.83 | 0.42 | -1.19 | 8.65 | -1.18 | 96.23 | -191.99 | 0.05 | -9.77 | 0.16 | -19.69 |
| | (0.32) | (0.33) | (0.47) | (0.29) | (0.34) | (0.31) | (0.00) | (0.00) | (0.00) | (0.00) | (0.00) | (0.00) |
| Japan | 0.01 | -0.34 | 0.86 | -0.29 | 39.38 | -0.23 | 90.89 | -208.23 | 0.05 | -10.19 | 0.13 | -17.85 |
| | (0.7) | (0.69) | (0.94) | (0.65) | (0.89) | (0.69) | (0.00) | (0.00) | (0.00) | (0.00) | (0.00) | (0.00) |
| China | 0.34 | -1.82 | 0.51 | -0.94 | 13.17 | -0.89 | | | | | | |
| | (0.00) | (0.00) | (0.00) | (0.00) | (0.00) | (0.00) | | | | | | |
| South Korea | 6.73 | -23.11 | 0.15 | -3.40 | 1.06 | -3.95 | 131.66 | -199.96 | 0.05 | -9.99 | 0.14 | -26.39 |
| | (0.08) | (0.04) | (0.04) | (0.04) | (0.04) | (0.04) | (0.00) | (0.00) | (0.00) | (0.00) | (0.00) | (0.00) |
| Indonesia | 0.59 | -3.36 | 0.36 | -1.22 | 7.26 | -1.17 | 113.81 | -185.58 | 0.05 | -9.59 | 0.19 | -23.67 |
| | (0.24) | (0.23) | (0.28) | (0.23) | (0.23) | (0.24) | (0.00) | (0.00) | (0.00) | (0.00) | (0.00) | (0.00) |
| Australia | 0.00 | 0.17 | 1.08 | 0.19 | 66.41 | 0.22 | 154.91 | -163.02 | 0.06 | -9.03 | 0.15 | -34.39 |
| | (0.71) | (0.75) | (0.99) | (0.78) | (0.97) | (0.78) | (0.00) | (0.00) | (0.00) | (0.00) | (0.00) | (0.00) |
