# Supplementary material for: COVID-19: Tail risk and predictive regressions
Source: PLoS One. 2022 Dec 1;17(12):e0275516. doi: 10.1371/journal.pone.0275516 (PMC9714707; doi:10.1371/journal.pone.0275516)
Supplement: S2 Table — (PDF) [file pone.0275516.s002.pdf]

Table S2. Wild bootstrap quasi-differenced unit root tests for Deaths based on Rademacher distribution with sieve based recolouring ($p-$values in brackets)

	$\Delta Deaths$						$\Delta^2 Deaths$					
	LR	MZ_α	MSB	MZ_t	MP_t	ADF	LR	MZ_α	MSB	MZ_t	MP_t	ADF
UK	0.38	-1.58	0.56	-0.89	15.55	-0.92	100.54	-189.85	0.05	-9.74	0.13	-20.69
	(0.35)	(0.39)	(0.53)	(0.34)	(0.45)	(0.35)	(0.00)	(0.00)	(0.00)	(0.00)	(0.00)	(0.00)
Germany	0.65	-2.44	0.43	-1.06	9.77	-1.19	116.42	-178.45	0.05	-9.43	0.16	-24.70
	(0.39)	(0.42)	(0.59)	(0.38)	(0.46)	(0.38)	(0.00)	(0.00)	(0.00)	(0.00)	(0.00)	(0.00)
France	1.70	-4.15	0.29	-1.22	6.22	-1.77	141.15	-165.54	0.05	-9.01	0.27	-30.95
	(0.1)	(0.17)	(0.13)	(0.22)	(0.18)	(0.12)	(0.00)	(0.00)	(0.00)	(0.00)	(0.00)	(0.00)
Italy	0.10	-0.92	0.59	-0.54	19.49	-0.54	119.48	-188.42	0.05	-9.69	0.15	-24.67
	(0.5)	(0.49)	(0.61)	(0.48)	(0.54)	(0.5)	(0.00)	(0.00)	(0.00)	(0.00)	(0.00)	(0.00)
Spain	4.49	-8.30	0.22	-1.84	3.69	-2.53						
	(0.01)	(0.04)	(0.03)	(0.07)	(0.07)	(0.01)						
Russia	0.00	0.28	1.19	0.33	82.22	0.52	110.55	-176.31	0.05	-9.39	0.14	-23.60
	(0.67)	(0.76)	(0.97)	(0.8)	(0.95)	(0.86)	(0.00)	(0.00)	(0.00)	(0.00)	(0.00)	(0.00)
Netherland	0.68	-2.87	0.42	-1.20	8.53	-1.22	120.78	-176.98	0.05	-9.41	0.14	-25.65
	(0.24)	(0.27)	(0.34)	(0.23)	(0.28)	(0.24)	(0.00)	(0.00)	(0.00)	(0.00)	(0.00)	(0.00)
Sweden	8.45	-10.10	0.22	-2.25	2.43	-3.21						
	(0.01)	(0.03)	(0.03)	(0.03)	(0.02)	(0.01)						
India	0.14	-0.74	0.80	-0.59	31.76	-0.60	142.49	-137.18	0.06	-8.28	0.18	-34.50
	(0.43)	(0.49)	(0.69)	(0.44)	(0.62)	(0.45)	(0.00)	(0)	(0.10)	(0)	(0)	(0.00)
Austria	0.57	-2.61	0.43	-1.13	9.35	-1.12	125.60	-165.39	0.05	-9.09	0.15	-27.69
	(0.4)	(0.43)	(0.57)	(0.38)	(0.46)	(0.4)	(0.00)	(0.00)	(0.00)	(0.00)	(0.00)	(0.00)
Finland	7.47	-14.26	0.19	-2.65	1.78	-3.33						
	(0)	(0.03)	(0.03)	(0.02)	(0.02)	(0.01)						
Ireland	2.72	-7.31	0.26	-1.91	3.35	-2.17	128.63	-161.97	0.06	-9.00	0.15	-28.66
	(0.07)	(0.06)	(0.08)	(0.05)	(0.05)	(0.05)	(0.00)	(0.00)	(0.00)	(0.00)	(0.00)	(0.00)
US	0.13	-0.86	0.75	-0.64	27.58	-0.61	81.99	-189.13	0.05	-9.72	0.13	-16.91
	(0.49)	(0.51)	(0.84)	(0.45)	(0.74)	(0.5)	(0.00)	(0.00)	(0.00)	(0.00)	(0.00)	(0.00)
Lithuania	0.18	-1.09	0.64	-0.70	20.83	-0.68	134.84	-137.92	0.06	-8.30	0.18	-32.56
	(0.54)	(0.6)	(0.85)	(0.55)	(0.72)	(0.55)	(0.00)	(0.00)	(0.00)	(0.00)	(0.00)	(0.00)
Canada	0.28	-1.36	0.59	-0.80	17.37	-0.83	135.84	-152.09	0.06	-8.71	0.17	-31.22
	(0.37)	(0.44)	(0.6)	(0.38)	(0.52)	(0.37)	(0.00)	(0.00)	(0.00)	(0.00)	(0.00)	(0.00)
Brazil	0.00	-0.75	0.52	-0.39	17.28	0.44	120.57	-167.56	0.05	-9.15	0.15	-26.41
	(0.69)	(0.51)	(0.57)	(0.53)	(0.51)	(0.84)	(0.00)	(0.00)	(0.00)	(0.00)	(0.00)	(0.00)
Mexico	1.18	-3.75	0.36	-1.37	6.53	-1.40	125.87	-158.55	0.06	-8.90	0.15	-28.35
	(0.18)	(0.22)	(0.32)	(0.19)	(0.22)	(0.22)	(0.00)	(0.00)	(0.00)	(0.00)	(0.00)	(0.00)
Argentina	4.83	-9.49	0.23	-2.17	2.63	-2.56						
	(0.03)	(0.05)	(0.05)	(0.05)	(0.05)	(0.05)						
Japan	0.52	-2.21	0.43	-0.96	10.42	-1.07	147.75	-156.80	0.06	-8.84	0.17	-33.43
	(0.4)	(0.44)	(0.57)	(0.4)	(0.46)	(0.39)	(0.00)	(0.00)	(0.00)	(0.00)	(0.00)	(0.00)
China	15.18	-44.19	0.11	-4.70	0.55	-5.97						
	(0.00)	(0.00)	(0.00)	(0.00)	(0.00)	(0.00)						
South Korea	1.03	-3.33	0.37	-1.23	7.32	-1.39	152.66	-152.24	0.06	-8.68	0.23	-33.43
	(0.2)	(0.27)	(0.31)	(0.25)	(0.27)	(0.2)	(0.00)	(0.00)	(0.00)	(0.00)	(0.00)	(0.00)
Indonesia	0.09	-0.95	0.54	-0.52	17.57	-0.54	121.94	-171.88	0.05	-9.24	0.19	-26.12
	(0.56)	(0.6)	(0.78)	(0.57)	(0.7)	(0.55)	(0.00)	(0.00)	(0.00)	(0.00)	(0.00)	(0.00)
Australia	1.61	-5.04	0.31	-1.59	4.86	-1.78	135.87	-160.31	0.06	-8.95	0.15	-30.43
	(0.23)	(0.21)	(0.22)	(0.2)	(0.2)	(0.21)	(0.00)	(0.00)	(0.00)	(0.00)	(0.00)	(0.00)
