# Supplementary material for: COVID-19: Tail risk and predictive regressions
Source: PLoS One. 2022 Dec 1;17(12):e0275516. doi: 10.1371/journal.pone.0275516 (PMC9714707; doi:10.1371/journal.pone.0275516)
Supplement: S3 Table — (PDF) [file pone.0275516.s003.pdf]

Table S3. Predictive regression tests for infection rates

	$\Delta Infections$						$\Delta^2 Infections$					
	T	q=4	q=8	q=12	q=16	HAC	T	q=4	q=8	q=12	q=16	HAC
UK FTSE 100	285	-0.18	1.59	0.45	0.45	-4.57***	284	-0.67	-0.43	-0.31	-0.74	1.92*
Germany DAX	288	-0.11	0.23	0.68	-0.03	-1.15	287	1.42	0.59	0.19	0.07	-0.15
France CAC 40	294	1.11	0.91	-0.11	0.20	-1.43	293	1.22	-0.10	-0.43	0.07	-0.79
Italy FTSE MIB	287	0.97	1.03	0.29	0.14	-2.78***	286	-1.11	-1.45	-0.07	-1.16	-2.24***
Spain IBEX 35	288	-1.35	1.39	1.02	2.45**	-1.12	287	0.70	-0.63	-0.79	0.01	-0.14
Russia MOEX	280	-0.99	-1.03	-1.09	-0.92	-3.48***	279	0.89	1.37	-0.62	1.28	-0.13
Netherland AEX	270	0.26	-0.23	-0.84	-1.05	-0.16	269	-0.60	-0.73	-0.95	-1.16	1.04
Sweden OMXS 30	281	0.43	-0.85	0.02	0.45	-1.51	280	-0.31	-0.85	-0.35	-1.77	-0.37
India SENSEX	280	0.86	1.44	1.43	-0.07	-4.25	279	0.34	-0.28	0.01	-0.01	-3.05***
Austria ATX	268	1.41	0.57	0.40	-0.75	-0.89	267	0.39	-0.44	0.75	0.06	1.07
Finland OMX Helsinki 25	284	1.17	0.18	0.70	0.58	-1.48	283	-0.91	-1.41	0.47	-0.48	-0.22
Ireland ISEQ	267	0.17	1.20	-1.74	-0.21	-1.75	266	-1.23	-0.94	-1.66	-1.43	-0.50
US Dow Jones	291	1.43	1.81	-0.17	-0.11	-2.67***	290	0.95	1.89	0.33	0.13	-1.90
US S&P 500	291	1.63	2.25	0.62	1.35	-2.67***	290	1.01	1.80	1.39	1.62	-1.94
Lithuania OMX Vilnius	264	1.23	0.24	0.70	0.83	-1.15	263	-0.51	-0.54	0.34	0.01	-1.54
Canada TSX	287	0.02	0.49	1.48	1.92	-3.53	286	3.07	1.26	1.53	1.04	0.73
Brazil iBovespa	259	-1.08	-1.21	-0.99	-0.85	-6.32***	258	0.40	0.60	0.14	1.10	-2.14**
Mexico IPC	263	-1.42	-1.56	-1.49	-1.05	-3.51	262	1.40	1.20	1.09	1.29	3.50
Argentina Merval	252	-0.85	0.92	-0.32	1.21	-1.73	251	0.64	-0.15	-0.90	-0.25	-3.14***
Japan NIKKEI 225	281	1.27	3.27***	1.12	1.18	-0.95	280	1.99	1.43	1.73	0.71	0.92
China SHANGHAI	277	0.77	-0.54	-0.22	1.34	-8.99***	276	0.81	1.28	2.29**	2.00	1.05
South KOSPI	284	0.91	1.02	1.06	1.40	-9.03***	283	1.34	1.94	0.70	0.73	0.20
Indonesia JCI	251	-0.95	-0.58	-0.25	-0.56	-1.15	250	-0.67	-0.37	0.40	0.30	-0.40
Australia ASX 50	290	-1.19	-0.91	1.08	-0.89	-2.52***	289	1.33	1.57	1.50	1.98	0.70
Australia ASX 200	290	-1.20	-0.91	1.12	-0.93	-2.52***	289	1.21	1.56	1.39	1.91	0.81
Australian All	290	-1.20	-0.95	1.02	-1.01	-2.52***	289	1.13	1.53	1.26	1.78	0.85
