# Supplementary material for: COVID-19: Tail risk and predictive regressions
Source: PLoS One. 2022 Dec 1;17(12):e0275516. doi: 10.1371/journal.pone.0275516 (PMC9714707; doi:10.1371/journal.pone.0275516)
Supplement: S4 Table — (PDF) [file pone.0275516.s004.pdf]

Table S4. Predictive regression tests for death rates

	$\Delta Deaths$						$\Delta^2 Deaths$					
	T	q=4	q=8	q=12	q=16	HAC	T	q=4	q=8	q=12	q=16	HAC
UK FTSE 100	260	0.33	-0.45	1.24	-0.40	-1.72	259	1.53	1.30	0.49	1.48	5.13***
Germany DAX	259	-0.87	-1.27	-1.24	-0.90	-0.55	258	-0.88	-0.84	-1.62	-1.05	-0.72
France CAC 40	278	2.37	0.53	1.43	-0.87	-0.83	277	0.81	0.37	0.48	-0.58	0.15
Italy FTSE MIB	272	-0.17	-0.54	0.71	0.33	-1.38	271	-0.90	-1.49	-0.74	-0.27	-0.24
Spain IBEX 35	267	1.05	0.37	1.31	1.24	-1.34	266	0.85	1.68	1.55	1.30	-0.99
Russia MOEX	248	-1.18	-1.15	-0.74	-0.24	-3.06	247	-1.17	0.21	0.16	0.88	-0.09
Netherland AEX	264	-0.73	-0.88	-1.68	-1.66	0.04	263	-0.74	-0.05	-0.90	-1.19	0.31
Sweden OMXS 30	255	-1.48	-1.68	-0.92	-1.60	1.29	254	-1.38	-1.48	0.19	-1.70	1.43
India SENSEX	253	0.24	1.25	1.34	1.48	-4.20***	252	1.50	-0.24	-0.27	0.87	-1.70
Austria ATX	256	-0.55	-1.48	-1.13	-0.68	1.43	255	-0.74	-1.03	-1.28	-0.34	3.08***
Finland OMX Helsinki 25	246	1.44	1.39	1.75	1.66	0.54	245	1.18	0.97	1.10	1.23	1.60
Ireland ISEQ	260	0.23	-0.32	0.39	-0.78	0.72	259	0.62	1.07	0.34	0.77	1.50
US Dow Jones	264	-0.47	-0.72	-0.17	0.41	-1.46	263	1.36	0.88	1.03	0.77	0.26
US S&P 500	264	0.19	0.26	0.26	0.67	-1.46	263	1.40	1.04	1.01	0.87	0.25
Lithuania OMX Vilnius	248	3.77	-0.55	-0.83	-0.46	1.50	247	-1.19	-0.11	-1.35	-0.50	1.23
Canada TSX	258	0.92	1.29	0.79	0.29	-2.96***	257	1.97	1.25	0.11	-0.49	0.21
Brazil iBovespa	246	-1.84	0.05	0.04	0.64	-3.31***	245	1.40	1.32	0.89	1.43	4.26***
Mexico IPC	250	-1.58	-1.89	-1.29	-1.75	-5.30***	249	0.51	1.46	0.94	-0.83	4.00***
Argentina Merval	248	-0.63	0.89	0.79	-0.81	-1.14	247	0.82	-0.44	1.04	1.36	-1.64
Japan NIKKEI 225	266	0.12	0.75	0.04	-0.25	-0.50	265	-0.94	-1.14	-1.67	-1.60	0.76
China SHANGHAI	277	0.01	1.52	-0.65	0.08	-6.25***	276	0.68	1.75	0.80	1.21	0.37
South KOSPI	265	-1.40	-0.79	-0.60	1.08	-6.25***	264	-1.11	0.11	0.48	0.29	12.03***
Indonesia JCI	244	-0.99	-0.81	-0.95	-1.22	-3.39***	243	-0.61	-0.47	-0.46	-0.94	-0.86
Australia ASX 50	266	-1.41	-1.06	-0.65	-0.94	-4.88***	265	1.22	1.51	1.55	1.81	-4.99***
Australia ASX 200	266	-1.39	-1.06	-0.52	-0.89	-4.82***	265	1.25	1.59	1.65	1.79	-5.02***
Australian All	266	-1.41	-1.07	-0.57	-0.91	-4.77***	265	1.27	1.60	1.67	1.77	-5.18***
