# Supplementary material for: COVID-19: Tail risk and predictive regressions
Source: PLoS One. 2022 Dec 1;17(12):e0275516. doi: 10.1371/journal.pone.0275516 (PMC9714707; doi:10.1371/journal.pone.0275516)
Supplement: S2 Fig — (PDF) [file pone.0275516.s006.pdf]

Fig S6. Hill's tail index estimates for positive changes in daily COVID-19 deaths

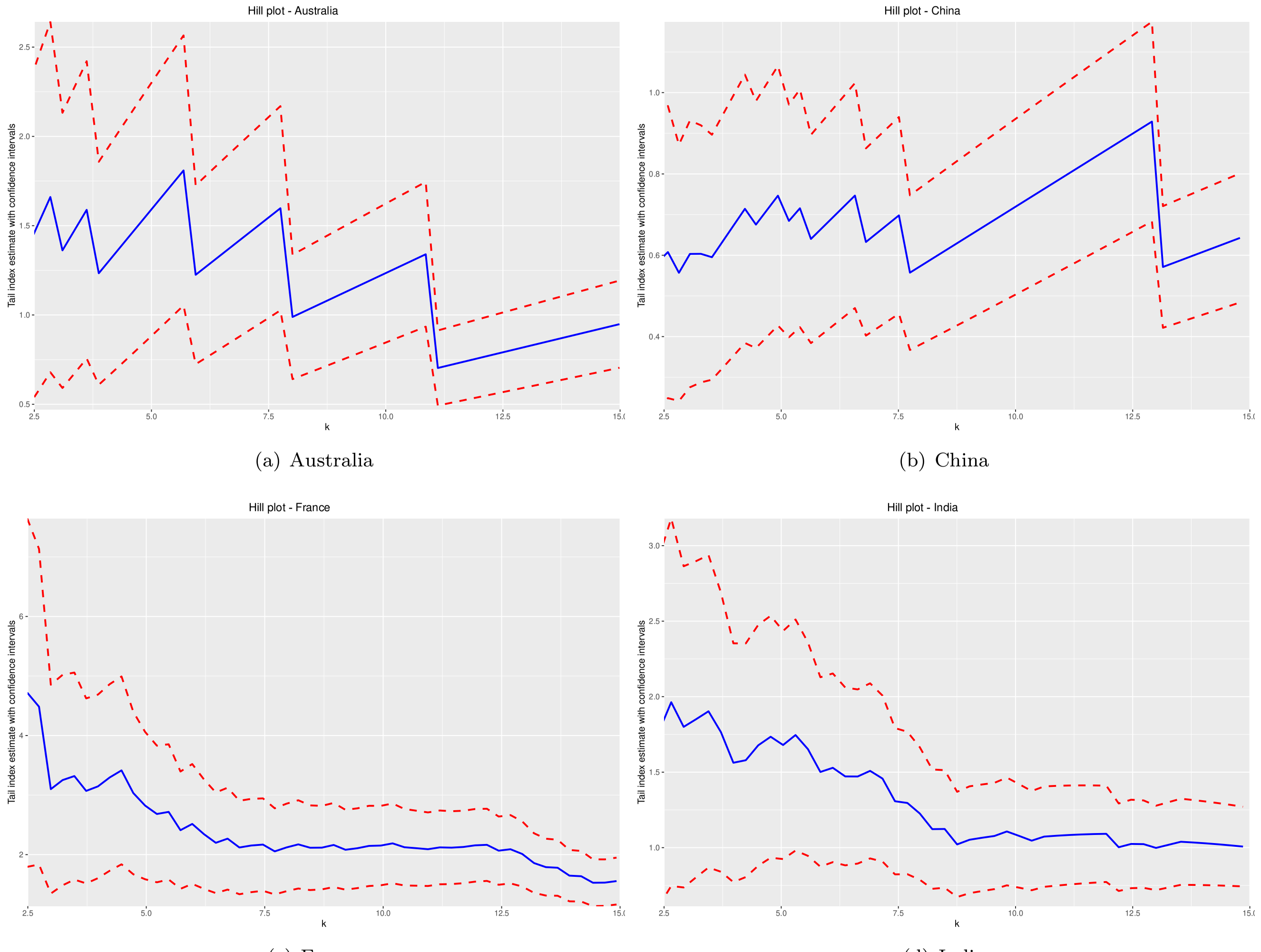

(a) Australia

(b) China

Fig S6. Hill's tail index estimates for positive changes in daily COVID-19 deaths (ctd)

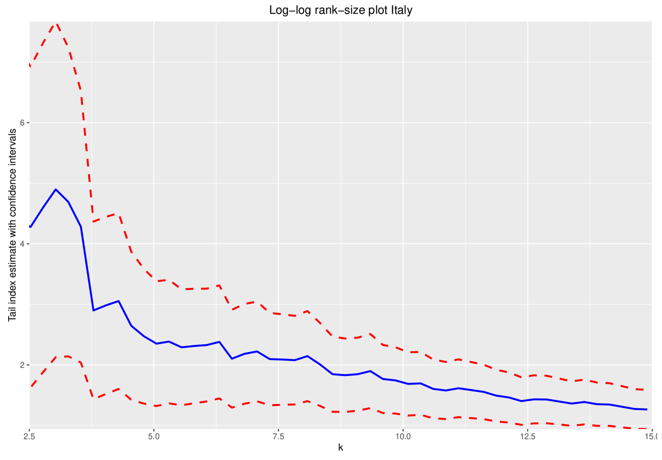

(e) Italy

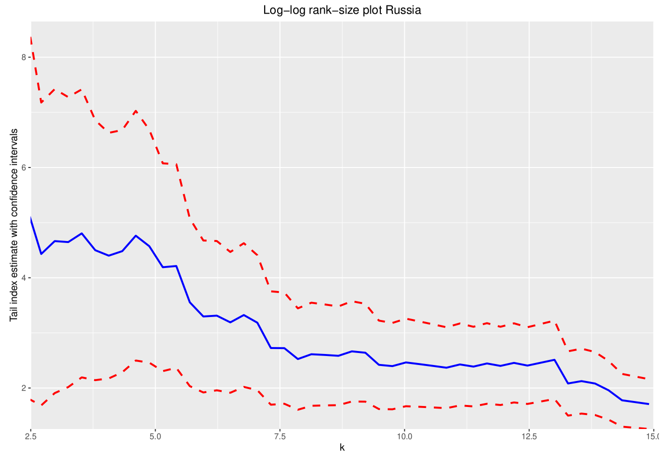

(f) Russia

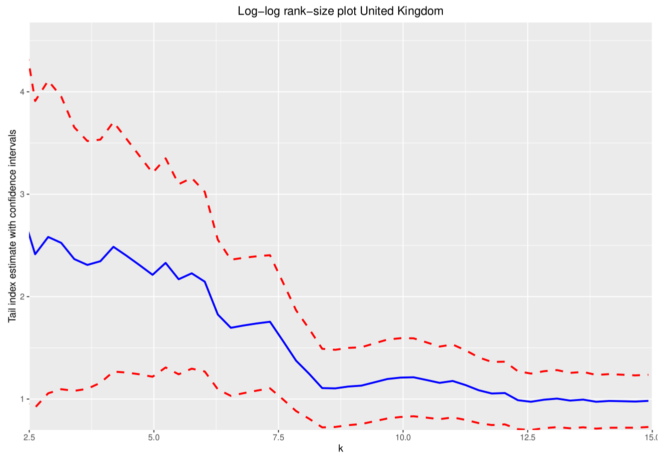

(g) UK

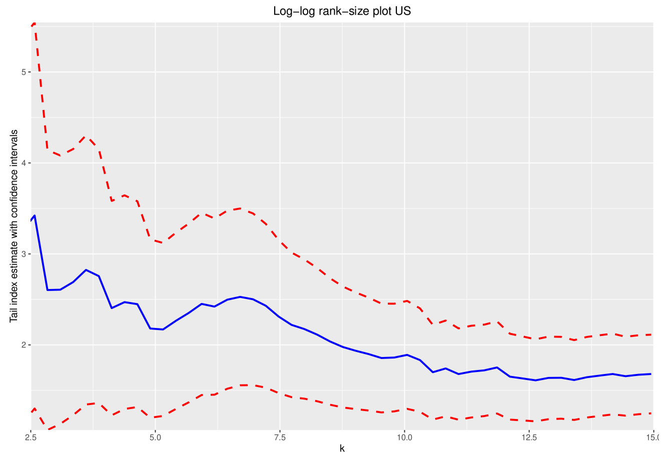

(h) US
