# Supplementary material for: COVID-19: Tail risk and predictive regressions
Source: PLoS One. 2022 Dec 1;17(12):e0275516. doi: 10.1371/journal.pone.0275516 (PMC9714707; doi:10.1371/journal.pone.0275516)
Supplement: S3 Fig — (PDF) [file pone.0275516.s007.pdf]

**Fig S7.** Log-log rank-size regression tail index estimates for positive changes in daily COVID-19 infections

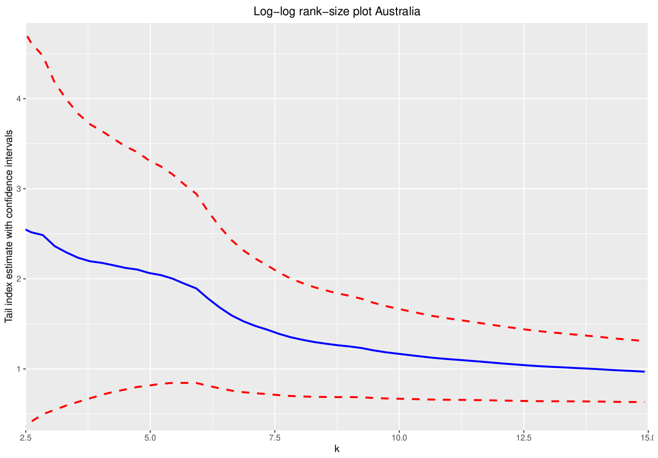


(a) Australia

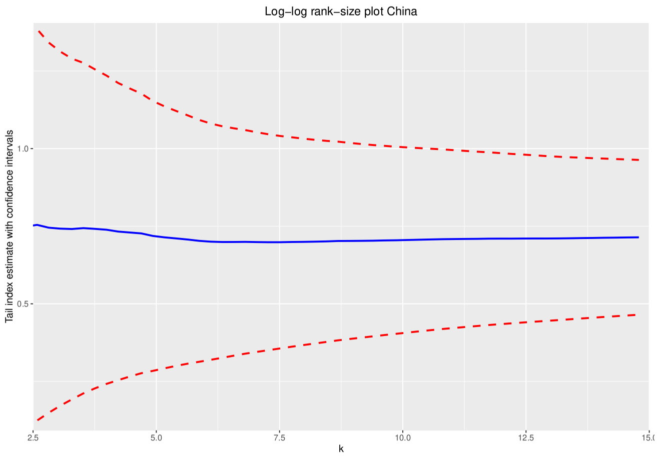


(b) China

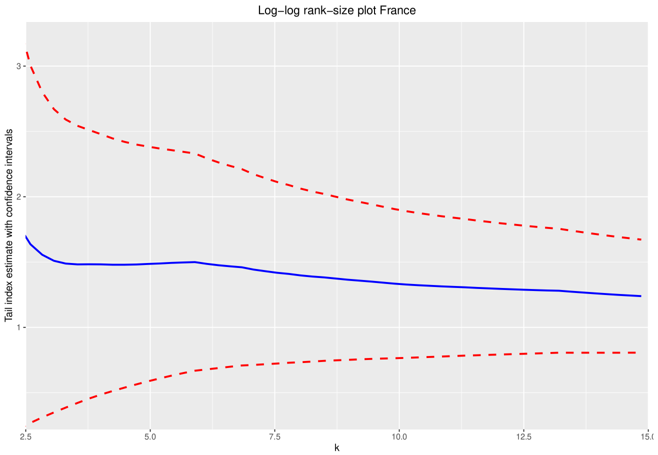


(c) [illegible]

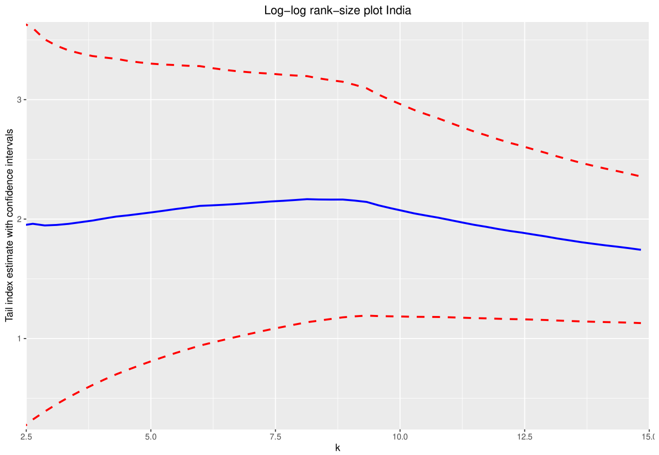


(d) [illegible]

**Fig S7.** Log-log rank-size regression tail index estimates for positive changes in daily COVID-19 infections (ctd)

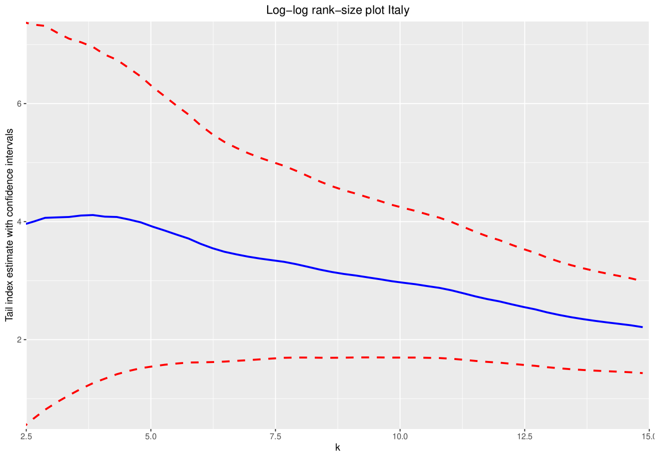


(e) Italy

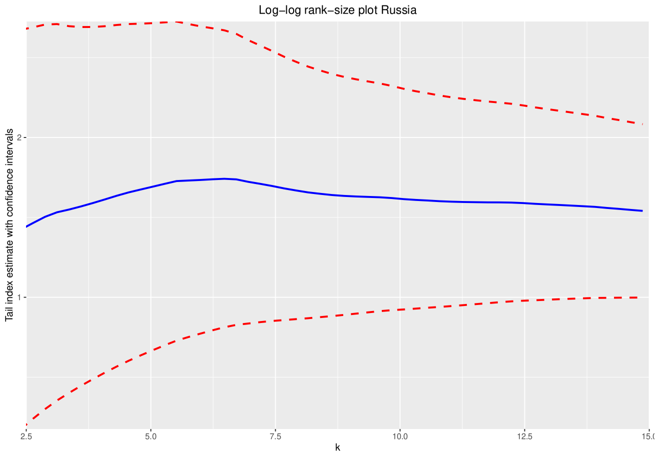


(f) Russia

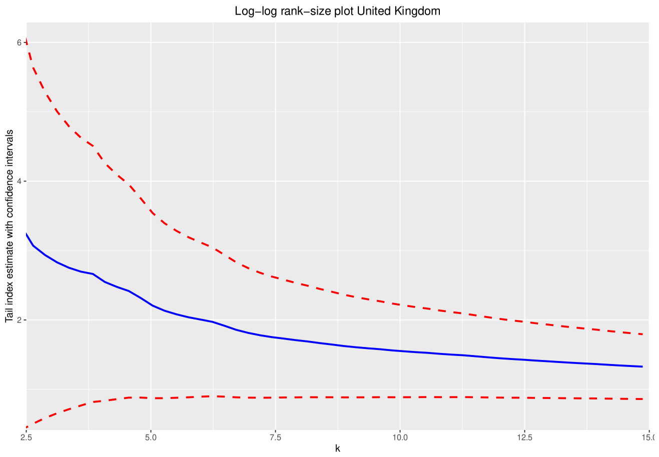


(g) UK

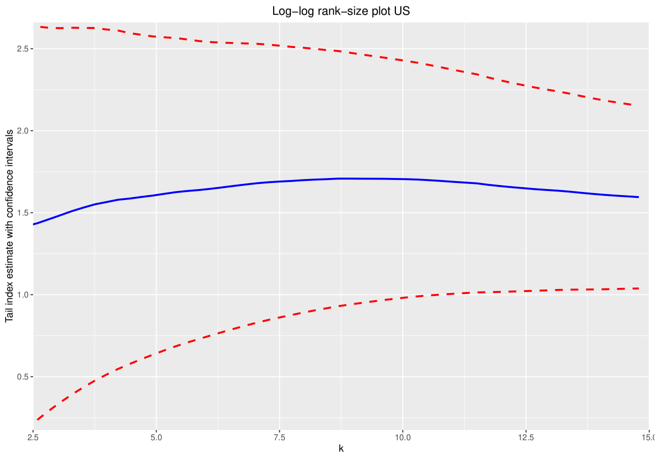


(h) US
